# Supporting cancer patients through the continuum of care: a view from the age of social networks and computer-mediated communication

**DOI:** 10.3747/co.v15i0.270

**Published:** 2008-08

**Authors:** J.L. Bender, L. O’Grady, A.R. Jadad

**Keywords:** Social support, supportive care, computer-mediated communication, social networks, Internet

## Abstract

Almost since its inception, the Internet has been used by ordinary people to connect with peers and to exchange health-related information and support. With the rapid development of software applications deliberately designed to facilitate social interaction, a new era is dawning in which patients and their loved ones can collaboratively build knowledge related to coping with illness, while meeting their mutual supportive care needs in a timely way, regardless of location. In this article, we provide background information on the use of “one-to-one” (for example, e-mail), “one-to-many” (for example, e-mail lists), and “many-to-many” (for example, message boards and chat rooms, and more recently, applications associated with Web 2.0) computer-mediated communication to nurture health-related social networks and online supportive care. We also discuss research that has investigated the use of social networks by patients, highlight opportunities for health professionals in this area, and describe new advances that are fuelling this new era of collaboration in the management of cancer.

